# Cable Bacteria and the Bioelectrochemical Snorkel: The Natural and Engineered Facets Playing a Role in Hydrocarbons Degradation in Marine Sediments

**DOI:** 10.3389/fmicb.2017.00952

**Published:** 2017-05-29

**Authors:** Bruna Matturro, Carolina Cruz Viggi, Federico Aulenta, Simona Rossetti

**Affiliations:** Water Research Institute, IRSA-CNRRome, Italy

**Keywords:** bioremediation, petroleum hydrocarbons biodegradation, next generation sequencing, oil spill snorkel, cable bacteria, sulfur cycle, marine sediment

## Abstract

The composition and metabolic traits of the microbial communities acting in an innovative bioelectrochemical system were here investigated. The system, known as Oil Spill Snorkel, was recently developed to stimulate the oxidative biodegradation of petroleum hydrocarbons in anoxic marine sediments. Next Generation Sequencing was used to describe the microbiome of the bulk sediment and of the biofilm growing attached to the surface of the electrode. The analysis revealed that sulfur cycling primarily drives the microbial metabolic activities occurring in the bioelectrochemical system. In the anoxic zone of the contaminated marine sediment, petroleum hydrocarbon degradation occurred under sulfate-reducing conditions and was lead by different families of *Desulfobacterales* (46% of total OTUs). Remarkably, the occurrence of filamentous *Desulfubulbaceae*, known to be capable to vehicle electrons deriving from sulfide oxidation to oxygen serving as a spatially distant electron acceptor, was demonstrated. Differently from the sediment, which was mostly colonized by *Deltaproteobacteria*, the biofilm at the anode hosted, at high extent, members of *Alphaproteobacteria* (59%) mostly affiliated to *Rhodospirillaceae* family (33%) and including several known sulfur- and sulfide-oxidizing genera. Overall, we showed the occurrence in the system of a variety of electroactive microorganisms able to sustain the contaminant biodegradation alone or by means of an external conductive support through the establishment of a bioelectrochemical connection between two spatially separated redox zones and the preservation of an efficient sulfur cycling.

## Introduction

Petroleum hydrocarbons are important sources of energy for daily life and industrial activities. During their production processes and/or transportation, tanker accidents may occur representing a global environmental issue (Prince, 1993). Oil spill is one of the major causes of marine pollution and represents a risk for human health and ecosystem functioning (Kvenvolden and Cooper, 2003; Van Hamme et al., 2003; Das and Chandran, 2011; Thapa et al., 2012; Sammarco et al., 2013).

In order to reduce the severe toxicity of these compounds, remediation strategies are urgently required. Technologies based on contaminant degradation processes operated by autochthonous microorganisms deserve increasing attention (Leahy and Colwell, 1990; Das and Chandran, 2011). Several studies exploited novel biological processes and investigated the ability of marine bacteria to mineralize these pollutants under sustainable conditions and at lower costs compared to physical-chemical treatments (Swannell et al., 1996; Van Hamme et al., 2003; Roling et al., 2004; Nikolopoulou et al., 2013). The success of hydrocarbon biodegradation depends on the environmental conditions favoring the action of specialized microorganisms. In particular, besides the adequate sources of nutrients (i.e., nitrogen, phosphorus, sulfur and iron), oxygen availability is fundamental for fast hydrocarbon biodegradation (even if anaerobic degradation may also occur at slower rates) (Ron and Rosenberg, 2014). To ensure the continuous availability of electron acceptors, remediation strategies based on the addition and/or the delivering of oxygen have been proposed (Zhang et al., 2010; Lu et al., 2014). However, due to its low solubility and fast reaction with reduced inorganic species (e.g., sulfide, ferrous ion), some of these strategies are often poorly effective and relatively expensive.

Promising alternatives based on bioelectrochemical systems were recently proposed for the clean up of contaminated marine environments, offering the opportunity to drive efficient and sustainable bioremediation processes employing electrodes as electron acceptors to stimulate the oxidation of petroleum-derived pollutants (Holmes et al., 2004; Zhang et al., 2010; Morris and Jin, 2012; Rakoczy et al., 2013; Cruz Viggi et al., 2015; Daghio et al., 2016). In some of these studies, the primary involvement of sulfur cycle on hydrocarbon degradation in bioelectrochemical systems was hypothesized (Cruz Viggi et al., 2015; Daghio et al., 2016). However, the role and identity of microorganisms responsible for such processes as well as the mechanisms involved were not deeply investigated and, in turn, fully understood. The occurrence of *Desulfobulbaceae* members on the anode surface and on the bulk of a bioelectrochemical system able to sustain toluene degradation was recently found even though its involvement was not directly proved (Daghio et al., 2016). The occurrence in natural environments of sulfate reducing bacteria belonging to *Desulfobulbaceae*, able to oxidize sulfide by using their ability to act as electron cables, was recently shown (Pfeffer et al., 2012). These microorganisms are capable to transport electrons, derived from sulfide oxidation, to oxygen as the final electron acceptor, using centimeter-long filaments as electrical cables.

In nature there is a great diversity of electroactive bacteria able to transfer electrons far beyond the cell surface to an electrode or vice versa (e.g., *Geobacter sulfurreducens, Acidithiobacillus ferrooxidans, Shewanella oneidensis)* (Rabaey and Rozendal, 2010; Liu et al., 2011; Rosenbaum et al., 2011; Levar et al., 2012; Bücking et al., 2013; Babauta et al., 2014; Dolch et al., 2014). Despite the metabolic potentialities of such microorganisms, only a few studies have dealt with electroactive bacteria in contaminated marine sediments where hydrocarbon bioelectrochemical degradation occurs (Holmes et al., 2004; Rowe et al., 2015).

In the present study, we have explored the structure and the associated metabolic traits of the microbial communities thriving in an innovative bioelectrochemical system (i.e., the Oil Spill Snorkel) recently developed for the anoxic biodegradation of petroleum hydrocarbons in marine sediments (Cruz Viggi et al., 2015). In the system, a graphite rod (i.e., the snorkel), half-buried within the anoxic contaminated sediment, was able to accept electrons deriving from the biological oxidation of contaminants and other reduced species in the marine sediment. In this configuration, electrons flow, along the conductive graphite rod, from the section of the rod buried in the anoxic sediment (i.e., anode) to the upper oxic section (i.e., cathode) where oxygen is reduced to form water in the presence of a catalyst (i.e., activated carbon). Overall, the system allows the formation of a (bio)electrochemical connection between the anoxic sediment and the overlying oxic water, thereby increasing the rate of oxidative reactions occurring in the sediment and positively affecting the extent of hydrocarbon degradation. Despite a higher degradation of total petroleum hydrocarbons was observed in the bioelectrochemical system compared to a control system, the interplay between the multiple biological reactions occurring in the Oil-Spill Snorkel, including hydrocarbon oxidation and sulfur cycle, was unresolved as well as the main mechanisms driving the impact, direct or indirect, of the electrode on the oxidation reactions.

## Materials and methods

### The oil-spill snorkel experiments

The “Oil-Spill Snorkel” experimental setup consisted of sacrificial microcosms containing crude oil-supplemented sandy sediment from Messina Harbor (Italy). The sediment was artificially contaminated in the laboratory with Intermediate Fuel Oil (IFO 180) to a final concentration of approximately 20 g/kg. Microcosms were prepared in 120-mL serum bottles. Each bottle was filled (starting from the bottom) with 50 grams of oil-supplemented sediment, 40 g of clean sand, 10 g of Norit® granular activated carbon (serving as oxygen reduction catalyst), and 40 mL of oxygenated seawater from the site. Graphite rods (1 or 3, depending on the treatment) were inserted vertically through the layers of the different materials to create the electrochemical connection between the anoxic sediment and the oxygenated overlaying water. Five different treatments were setup, namely: treatment “S^3^” which contained 3 graphite rods, treatment “S” which contained 1 graphite rod, treatment “C” (biotic control) which contained no graphite rods, treatment “B^3^” (autoclaved control) which contained 3 graphite rods and was autoclaved (120°C for 1 h) on 3 successive days and treatment “B” (autoclaved control) which contained 1 graphite rods and was also autoclaved (120°C for 1 h) on 3 successive days.

Once prepared all the microcosms were statically incubated in the dark in a temperature-controlled room at 20 ± 1°C. Weekly, the headspace of the bottles was analyzed for oxygen consumption and carbon dioxide evolution by gas-chromatography (GC) with thermal conductivity detector (TCD). At fixed times, one bottle from each treatment was sacrificed: the sediment was analyzed (upon liquid–solid extraction) by GC and flame ionization detector (FID) for quantification of total petroleum hydrocarbons (TPH); the liquid phase was analyzed by ion chromatography (IC) for quantification of seawater anions.

### CARD-FISH

Sediment samples and biofilms growing on electrode surface were collected at the end of the treatment “S^3^” (*t* = 417 d) for CARD-FISH analysis. 1 g of marine sediment was fixed in formaldehyde and processed as previously reported (Cruz Viggi et al., 2015). In parallel, microorganisms on the electrode surface were scraped with a sterile spatula, dissolved in PBS buffer with formaldehyde (2% v/v). Microorganisms detached both from the marine sediment particles and from the electrode surface were filtered through 0.2 μm polycarbonate filters (Ø 47 mm, Millipore) by gentle vacuum (<0.2 bar) and stored at −20°C until use. Each sample has been used for Catalyzed Reporter Deposition-Fluorescence *In situ* Hybridization (CARD-FISH) following the procedure published elsewhere (Matturro et al., 2016a). Oligonucleotide probes targeting *Deltaproteobacteria* (DELTA495abc; Loy et al., 2002) and *Desulfobulbaceae* (DSB706; Schauer et al., 2014) were employed following the hybridization conditions reported elsewhere (Matturro et al., 2016a). The analysis was performed by epifluorescence microscopy (Olympus, BX51). Images were captured with *Olympus F-View CCD* camera and handled with *Cell^F* software (Olympus, Germany).

### DNA extraction

DNA extraction for NGS analysis was performed on samples collected at the end of the treatment “S^3^” (*t* = 417 days). In detail, 0.25 g of dry marine sediment were collected with a sterile spatula and processed for DNA extraction with *Power Soil DNA extraction kit* (*MoBio, Italy*) following the manufacturer's instructions. Simultaneously, biofilm growing on the electrode surface was gently scraped with a sterile spatula and dissolved in 15 mL sterile *Milli-Q* water (*Millipore, Italy*). Pellet was collected after 15 min of centrifugation at 15,000 g and processed for DNA extraction with Power Soil DNA extraction kit (*MoBio, Italy*) following the manufacturer's instructions.

Purified DNA from each sample was eluted in 100 μL sterile *Milli-Q* water and 10 ng of extracted DNA was used for the following NGS analysis.

### Next generation sequencing (NGS)

16S rRNA Amplicon Library Preparation (V1–3) was performed as detailed in Matturro et al. (2016b). The procedure for bacterial 16S rRNA amplicon sequencing targeting the V1–3 variable regions is based on Caporaso et al. (2012), using primers adapted from the Human Gut Consortium (Ward et al., 2012). 10 ng of extracted DNA was used as template in the PCR reaction (25 μL) containing dNTPs (400 nM of each), MgSO4 (1.5 mM), Platinum® Taq DNA polymerase HF (2 mU), 1X Platinum® High Fidelity buffer (Thermo Fisher Scientific, USA) and barcoded library adaptors (400 nM) containing V1–3 primers (27F: 5′-AGAGTTTGATCCTGGCTCAG-3′; 534R: 5′-ATTACCGCGGCTGCTGG-3′). All PCR reactions were run in duplicate and pooled afterward. The amplicon libraries were purified using the Agencourt® AMpure XP bead protocol (Beckmann Coulter, USA). Library concentration was measured with Quant-iTTM HS DNA Assay (Thermo Fisher Scientific, USA) and quality validated with a Tapestation 2200, using D1K ScreenTapes (Agilent, USA).

The purified sequencing libraries were pooled in equimolar concentrations and diluted to 4 nM. The samples were paired end sequenced (2 × 301 bp) on a MiSeq (Illumina) using a MiSeq Reagent kit v3, 600 cycles (Illumina) following the standard guidelines for preparing and loading samples on the MiSeq. 10% Phix control library was spiked in to overcome low complexity issue often observed with amplicon samples.

Forward and reverse reads were trimmed for quality using Trimmomatic v. 0.32 (Bolger et al., 2014) with the settings SLIDINGWINDOW:5:3 and MINLEN:275 and merged using FLASH v. 1.2.7 (Magoč and Salzberg, 2011) with the settings -m 25 -M 200. The merged reads were dereplicated, formatted for use in the UPARSE workflow (Edgar, 2013) and clustered using the usearch v. 7.0.1090 -cluster_otus command with default settings. OTU abundances were estimated using the usearch v. 7.0.1090 -usearch_global command with -id 0.97. Taxonomy was assigned using the RDP classifier (Wang et al., 2007) as implemented in the parallel_assign_taxonomy_rdp.py script in QIIME (Caporaso et al., 2010), using the MiDAS database v.1.20 (McIlroy et al., 2015). The results were analyzed in R (R Core Team, 2015) through the Rstudio IDE using the ampvis package v.1.9.1 (Albertsen et al., 2015).

### Biodiversity indices

Evenness (E), Shannon (H) and the taxonomic distinctness (TD) indices were used to describe the biodiversity of the marine sediment and the biofilm on the electrode surface by using Past version 3.10.

## Results

### Effect of the snorkel on key biogeochemical processes in oil-contaminated sediments

The crude oil-supplemented microcosms containing 3 graphite rods (i.e., treatment “S^3^”) displayed a 1.7-fold higher cumulative oxygen uptake and a 1.4-fold higher cumulative CO_2_ evolution compared to the snorkel-free biotic controls (Cruz Viggi et al., 2015). In agreement with that, the initial rate of petroleum hydrocarbons biodegradation was also substantially enhanced. Indeed, while after 200 days of incubation a negligible degradation of hydrocarbons was noticed in snorkel-free control microcosms, a substantial reduction of 12 and 21% was observed in microcosms containing 1 and 3 snorkels, respectively. Following a more prolonged incubation (day 417), an extensive degradation of TPH occurred in all treatments, including the autoclaved controls, with removals exceeding 80% in most treatments. Sulfate reduction fuelled by TPH and/or by the organic matter contained in the sediment was observed in all biotic treatments, although it proceeded at a substantially higher rate in the snorkel-free controls (Cruz Viggi et al., 2015).

A possible explanation for that is the preferential use of the “snorkel” over sulfate as respiratory electron acceptor for the oxidation of organic substrates in the sediment. On the other hand, another possible explanation is that the “snorkels” facilitated the (biotic or abiotic) back-oxidation (to sulfate) of the sulfide generated in the sediment from the activity of sulfate-reducing microorganisms, hence resulting in an apparently lower sulfate reduction.

### Microscopic analysis

The predominance of members of *Proteobacteria* both in the bulk sediment and on the electrode surface of the Oil-Spill Snorkel microcosms (treatment “S^3^”) was shown by CARD-FISH analysis. In particular, members of *Deltaproteobacteria* and *Chloroflexi* were predominant in the initial contaminated marine sediment and increased up to 9.8 and 8.6-fold respectively at the end of the treatment (Cruz Viggi et al., 2015). On the contrary, the electrode surface was mostly colonized (~95% of total bacteria) by cells belonging to *Alphaproteobacteria, Gammaproteobacteria*, and to lesser extent by *Deltaproteobacteria* evidencing the existence on the electrode surface of a distinct microbial niche (Cruz Viggi et al., 2015).

**Figure 1:**
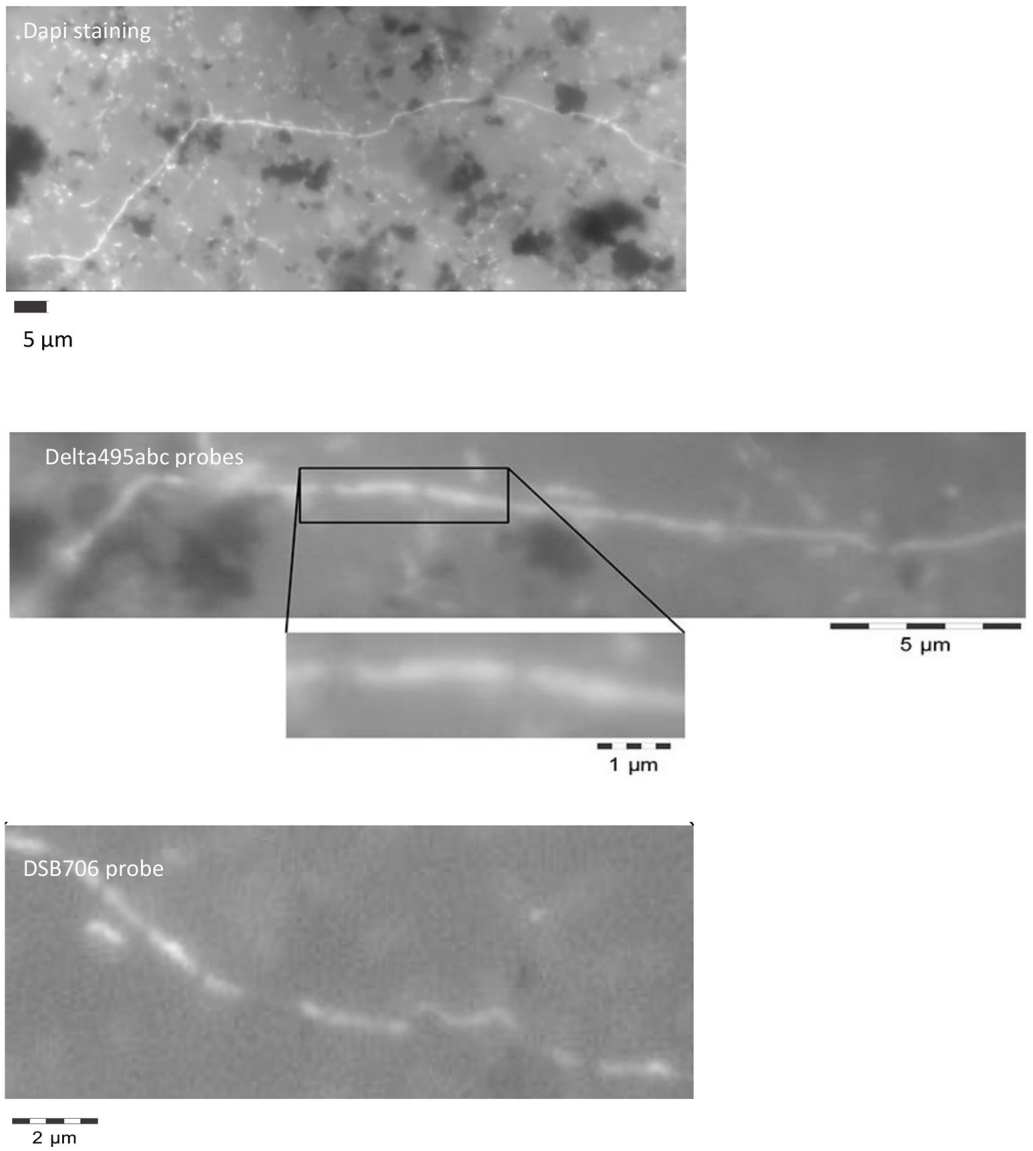
Filamentous *Desulfobulbaceae* evidenced by microscopic analysis at the end of the treatment in the marine sediment after DAPI staining and CARD-FISH analysis with oligonucleotide probes targeting *Deltaproteobacteria* (Delta495abc probes) and *Desulfobulbaceae* (DSB706 probe).

Interestingly, the microscopic analysis revealed mainly in the bulk sediment the presence of filamentous bacteria belonging to *Deltaproteobacteria* (Figure 1). The filaments were composed of bacilli with clear indentations at septa and a total length ranging from 10 to 100 μm. However, the actual length of the filaments in the original sediment before the sample pretreatment required for the CARD-FISH assay (i.e., vortexing to detach cells from sediment particles and successive centrifugation to separate cells from the sediment particles) was probably higher. As shown in Figure 1, the filamentous bacteria and some single rod-shaped cells positively hybridized with DSB706 probe specific for *Desulfobulbaceae* family.

### Microbiome of the contaminated marine sediment

**Figure 2:**
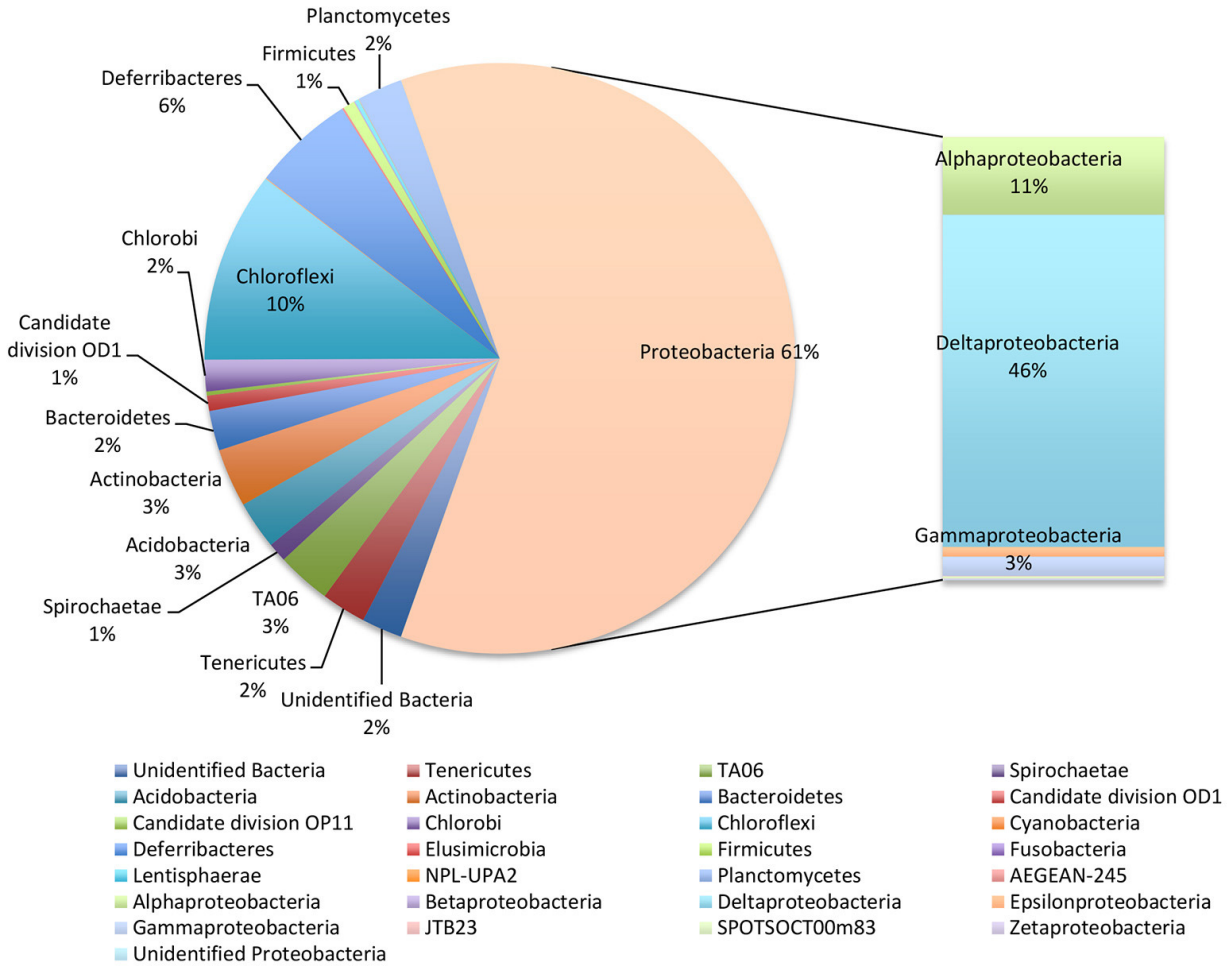
Microbiome of the bulk sediment. Data are reported as percentage out of total OTUs produced by NGS analysis.

NGS analysis of the bulk sediment produced a total of 256 OTUs. *Proteobacteria* dominated the microbiome representing 61% of total OTUs. In particular, *Deltaproteobacteria* members were the most abundant within the entire microbiome (46%) and were mostly affiliated to *Desulfobacteraceae* (19.6%), *Desulfobulbaceae* (13.5%) and *Desulfarculaceae* (10%) (Figure 2, Table 1). Further, *Chloroflexi* represented 10% of total OTUs and were mostly affiliated to *Anaerolineaceae* family, while *Alphaproteobacteria* members (11% of total OTUs) were related to *Rhodospirillaceae* family, including *Magnetovibrio, Pelagibius, Thalassospira*, and *Defluviicoccus* genera, and to *Rhodobacteraceae* family (Figure 2, Table 1). Additionally, members of *Deferribacteres* phylum were abundant and represented 6% of total OTUs of which SAR406 clade (Marine group A) members were the most representative ones (Figure 2, Table 1).

**Table 1:** Phylogenetic affiliation of the most representative OTUs detected by NGS in the marine sediment.

**% OTUs**	**Order**	**Family**	**Genus**	***n*° OTUs**
***Deltaproteobacteria***				
12.87	Desulfobacterales	Desulfobulbaceae	Uncultured	3
0.56	Desulfobacterales	Desulfobulbaceae	Desulfobulbus	2
0.16	Desulfobacterales	Desulfobulbaceae	MSBL7	2
10.35	Desulfobacterales	Desulfobacteraceae	Uncultured	8
4.87	Desulfobacterales	Desulfobacteraceae	Desulfotignum	2
2.27	Desulfobacterales	Desulfobacteraceae	Desulfosarcina	1
1.45	Desulfobacterales	Desulfobacteraceae	SEEP-SRB1	1
0.76	Desulfobacterales	Desulfobacteraceae	Desulfosarcina	1
0.07	Desulfobacterales	Nitrospinaceae	Nitrospina	1
10.35	Desulfarculales	Desulfarculaceae	Uncultured	3
0.54	Desulfuromonadales	Sva1033	Uncultured	2
0.73	FW113	Unidentified	Unidentified	1
0.12	Myxococcales	0319-6G20	Unidentified	1
0.43	SAR324 clade (Marine group B)	Unidentified	Unidentified	1
0.09	Sh765B-TzT-29	Unidentified	Unidentified	1
***Chloroflexi***				
9.20	Anaerolineae	Anaerolineales	Anaerolineaceae	18
0.15	Anaerolineae	Anaerolineales	Anaerolineaceae	1
0.46	Anaerolineae	Anaerolineales	Anaerolineaceae	2
0.01	Dehalococcoidia	Dehalococcoidales	Incertae Sedis	1
0.16	Dehalococcoidia	Dehalococcoidales	Dehalococcoidaceae	1
0.34	Dehalococcoidia	MSBL5	Unidentified	2
0.01	KD4-96	Uncultured	Unidentified	1
0.11	Dehalococcoidia	Uncultured	Unidentified	1
***Alphaproteobacteria***				
1.97	Rhodospirillales	Rhodospirillaceae	Magnetovibrio	4
0.93	Rhodospirillales	Rhodospirillaceae	Uncultured	16
0.78	Rhodospirillales	Rhodospirillaceae	Pelagibius	2
0.48	Rhodospirillales	Rhodospirillaceae	Thalassospira	2
0.45	Rhodospirillales	Rhodospirillaceae	Nisaea	1
0.39	Rhodospirillales	Rhodospirillaceae	Defluviicoccus	3
0.06	Rhodospirillales	Rhodospirillaceae	Magnetospira	3
0.04	Rhodospirillales	Rhodospirillaceae	OM75 clade	1
0.83	Rhodobacterales	Rhodobacteraceae	Roseovarius	2
0.11	Rhodobacterales	Rhodobacteraceae	Uncultured	4
0.06	Rhodobacterales	Rhodobacteraceae	Loktanella	1
0.02	Rhodobacterales	Rhodobacteraceae	Pacificibacter	1
0.01	Rhodobacterales	Rhodobacteraceae	Ruegeria	1
0.31	Rhizobiales	Methylobacteriaceae	Methylobacterium	3
0.13	Rhizobiales	Bradyrhizobiaceae	Uncultured	1
0.11	Rhizobiales	Rhodobiaceae	Uncultured	1
0.06	Rhizobiales	Bradyrhizobiaceae	Bosea	1
0.05	Rhizobiales	Bradyrhizobiaceae	Rhodopseudomonas	1
0.05	Rhizobiales	Rhodobiaceae	Rhodobium	1
0.04	Rhizobiales	Rhizobiaceae	Rhizobium	1
0.70	4-Org1-14	Uncultured	Uncultured	4
0.29	Caulobacterales	Caulobacteraceae	Caulobacter	1
0.21	Caulobacterales	Caulobacteraceae	Brevundimonas	1
0.18	DB1-14	Uncultured	Uncultured	1
0.01	Kordiimonadales	Kordiimonadaceae	Kordiimonas	1
0.41	Parvularculales	Parvularculaceae	Parvularcula	4
***Deferribacteres***				
3.98	Deferribacterales	SAR406 clade (Marine group A)	Unidentified	3
0.85	Deferribacterales	Deferribacterales Incertae Sedis	Caldithrix	1
0.04	Deferribacterales	PAUC34f	Unidentified	1
0.65	Deferribacterales	LCP-89	Unidentified	3
***Tenericutes***				
2.45	Mollicutes	NB1-n	Unidentified	3
**TA06**				
2.90	Unidentified	Unidentified	Unidentified	5
***Gammaproteobacteria***				
0.88	Incertae Sedis	Unknown family	Sedimenticola	1
0.76	Xanthomonadales	JTB255 marine benthic group	Unidentified	2
0.40	Pseudomonadales	Moraxellaceae	Acinetobacter	2
0.17	Unidentified	Unidentified	Unidentified	4
0.16	NKB5	Unidentified	Unidentified	1
0.06	34P16	Unidentified	Unidentified	1
0.06	Chromatiales	Ectothiorhodospiraceae	Acidiferrobacter	1
0.06	Oceanospirillales	Unidentified	Unidentified	1
0.06	Order Incertae Sedis	Family Incertae Sedis	Marinicella	1
0.02	Alteromonadales	Alteromonadaceae	Marinobacter	1
0.01	Chromatiales	Unidentified	Unidentified	1
0.01	PYR10d3	Unidentified	Unidentified	1

### Microbiome of the biofilm growing at the surface of the electrode

**Figure 3:**
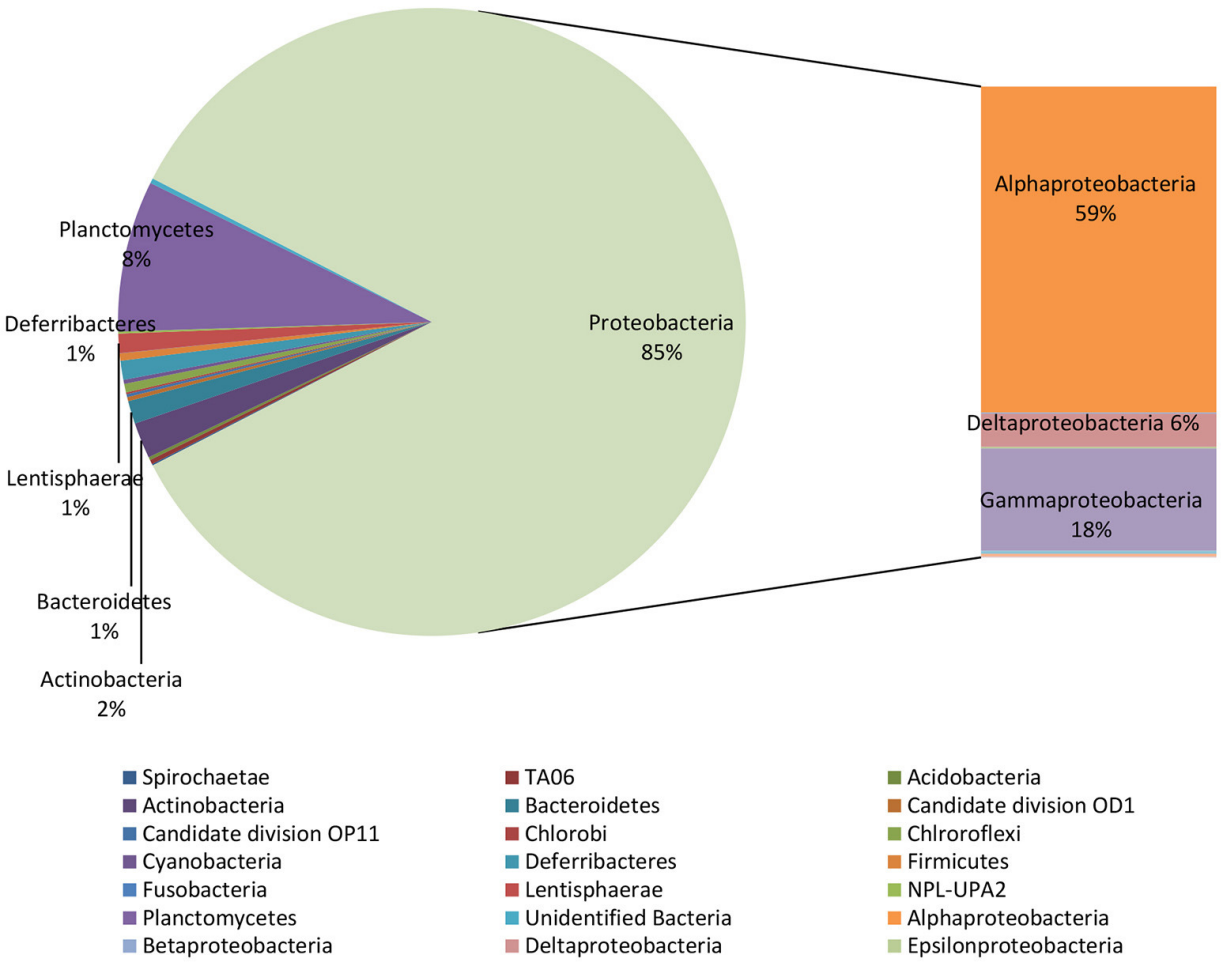
Microbiome of the biofilm taken from the electrode surface. Data are reported as percentage out of total OTUs produced by NGS analysis.

NGS analysis, conducted on the biofilm sample taken from the surface of the electrode buried within the sediment, provided 240 OTUs. *Proteobacteria* represented 85% of total OTUs and, diversely from the marine sediment, mainly comprised of *Alphaproteobacteria* (59% of total OTUs) (Figure 3). They were mostly affiliated to *Rhodospirillaceae* family (33%), including *Magnetovibrio* (11%), *Thalassospira* (5%), *Pelagibus* (3%), *Nisaea* (3%), and *Defluviicoccus* (3.5%) genera. As shown in Table 2, 6% of total OTUs within *Rhodospirillaceae* family were unidentified. Further, *Gammaproteobacteria* members (18%), affiliated to *Sedimenticola* (9.6%), and *Xanthomonadales* (4%), and *Deltaproteobacteria* (6%), mostly represented by *Desulfobulbaceae*, were also found on the electrode surface. Moreover, 8% of total OTUs were affiliated to *Planctomycetes*, whose most representative members belonged to *Phycisphaeraceae* family (SM1A02 genus) (Figure 3, Table 2).

**Table 2:** Phylogenetic affiliation of the most representative OTUs detected by NGS in the biofilm taken from electrode surface.

**% OTUs**	**Order**	**Family**	**Genus**	***n*° OTUs**
***Alphaproteobacteria***				
10.8	Rhodospirillales	Rhodospirillaceae	Magnetovibrio	4
5.92	Rhodospirillales	Rhodospirillaceae	Uncultured	10
5.16	Rhodospirillales	Rhodospirillaceae	Thalassospira	3
4.67	Rhodospirillales	Rhodospirillaceae	Unidentified	10
3.46	Rhodospirillales	Rhodospirillaceae	Defluviicoccus	3
3.32	Rhodospirillales	Rhodospirillaceae	Pelagibus	2
3.04	Rhodospirillales	Rhodospirillaceae	Nisaea	1
0.58	Rhodospirillales	Rhodospirillaceae	Magnetospira	4
0.46	Rhodospirillales	Rhodospirillaceae	OM75 clade	1
0.03	Rhodospirillales	AT-s3-44	Unidentified	1
6.23	4-Org1-14	Unidentified	Unidentified	4
0.27	Rhodobacterales	Rhodobacteraceae	Roseovariua	2
0.10	Rhodobacterales	Rhodobacteraceae	Unidentified	4
0.10	Rhodobacterales	Rhodobacteraceae	Pacificibacter	1
0.09	Rhodobacterales	Rhodobacteraceae	Rhodovulum	1
0.03	Rhodobacterales	Rhodobacteraceae	Paracoccus	1
0.56	Caulobacterales	Caulobacteraceae	Brevundimonas	1
0.15	Caulobacterales	Caulobacteraceae	Caulobacter	1
0.02	Caulobacterales	Hyphomonadaceae	Unidentified	1
2.11	DB1-14	Unidentified	Unidentified	2
0.15	Kordiimonadales	Kordiimonadaceae	Kordiimonas	1
0.11	OCS116 clade	Unidentified	Unidentified	2
0.89	Parvularculales	Parvularculaceae	Parvularcula	4
0.84	Rhizobiales	Methylobacteriaceae	Methylobacterium	4
0.78	Rhizobiales	Rhodobiaceae	Uncultured	1
0.20	Rhizobiales	Bradyrhizobiaceae	Unidentified	2
0.10	Rhizobiales	Bradyrhizobiaceae	Rhodopseudomonas	1
0.07	Rhizobiales	Bradyrhizobiaceae	Bosea	1
0.06	Rhizobiales	Rhizobiaceae	Rhizobium	1
0.03	Rhizobiales	Rhizobiales Incertae Sedis	Bauldia	1
0.01	Rhizobiales	Rhodobiaceae	Rhodobium	1
0.11	Rickettsiales	SHWN-night2	Unidentified	2
0.11	Rickettsiales	SHWN-night2	Unidentified	2
0.04	Rickettsiales	Mitochondria	Unidentified	1
0.04	Rickettsiales	Mitochondria	Unidentified	1
0.01	Rickettsiales	T9d	Unidentified	1
1.66	Sneathiellales	Sneathiellaceae	Sneathiella	2
0.89	Sphingomonadales	Sphingomonadaceae	Unidentified	1
0.19	Sphingomonadales	Sphingomonadaceae	Sphingomonas	2
0.10	Sphingomonadales	Unidentified	Unidentified	1
0.03	Sphingomonadales	Erythrobacteraceae	Unidentified	1
5.05	unidentified	Unidentified	Unidentified	5
***Gammaproteobacteria***				
9.63	Incertae Sedis	Unknown family	Sedimenticola	1
3.83	Xanthomonadales (Marine group B)	JTB255		3
2.52	Pseudomonadales	Moraxellaceae	Acinetobacter	2
0.71	34P16	Unidentified	Unidentified	1
0.47	Oceanospirillales	Unidentified	Unidentified	1
0.46	unidentified	Unidentified	Unidentified	4
0.22	NKB5	Unidentified	Unidentified	1
0.21	PYR10d3	Unidentified	Unidentified	1
0.14	Chromatiales	Unidentified	Unidentified	1
0.09	Alteromonadales	Alteromonadaceae	Marinobacter	1
0.04	Incertae Sedis	Incertae Sedis	Marinicella	2
0.02	Chromatiales	Granulosicoccaceae	Granulosicoccus	1
0.02	Chromatiales	Ectothiorhodospiraceae	Acidiferrobacter	2
***Planctomycetes***				
6.91	Phycisphaerales	Phycisphaeraceae	SM1A02	6
0.30	OM190	Unidentified	Unidentified	3
0.14	BD7-11	Unidentified	Unidentified	1
0.05	Phycisphaerales	Phycisphaeraceae	Phycisphaera	1
0.35	Unidentified	Unidentified	Unidentified	1
***Deltaproteobacteria***				
3.22	Desulfobacterales	Desulfobulbaceae	Unidentified	1
0.52	Desulfobacterales	Desulfobulbaceae	Desulfobulbus	2
0.09	Desulfobacterales	Desulfobulbaceae	MSBL7	2
0.06	Desulfobacterales	Desulfobacteraceae	Desulfosarcina	2
0.04	Desulfobacterales	Desulfobacteraceae	Desulfotignum	1
0.13	Desulfobacterales	Desulfobacteraceae	Uncultured	2
0.10	Desulfobacterales	Desulfobacteraceae	Unidentified	2
0.65	Desulfobacterales	Nitrospinaceae	Nitrospina	1
0.58	Myxococcales	0319-6G20	Unidentified	1
0.34	Sh765B-TzT-29	Unidentified	Unidentified	1
0.02	SAR324 clade (Marine group B)		Unidentified	1
0.20	Unidentified	Unidentified	Unidentified	1

### Biodiversity

The analysis of bacterial diversity was performed from data generated by NGS. Overall, all indices (TD, H, E) indicated a higher biodiversity in the sediment compared to the biofilm attached on the electrode surface. Values of TD, which captures phylogenetic diversity and it is more closely linked to functional diversity (Clarke and Warwick, 1999), were low indicating the occurrence of distinct microbial niches occurring in the sediment and on the electrode surface (Table 3). Similarly, S and H indices were low in both matrixes analyzed.

**Table 3:** Biodiversity indices of the microbial community inhabiting the marine sediment and the biofilm growing at the electrode surface.

**Biodiversity index**	**Sediment**	**Electrode**
Taxonomic distinctness	1.5	1.3
Shannon	2.08	1.54
Evenness	0.35	0.23

## Discussion

### Microbiome of the marine sediment

At the end of the treatment, the contaminated marine sediment was mostly composed by members of *Desulfobulbaceae* (14% of total OTUs), *Desulfobacteraceae* (12% of total OTUs), and *Desulfarculaceae* (10% of total OTUs). The majority of the representatives of these OTUs still results uncultured and comprises strictly anaerobic sulfate reducing bacteria able to reduce sulfate, sulfite and thiosulfate to sulfide, consistently with the occurrence of sulfate-reduction, possibly fuelled by TPH, in the Oil-Spill Snorkel treatments. Indeed, these microorganisms have been already found in marine habitats, isolated from oil-reservoir and/or marine environments and reported as sulfate-reducing hydrocarbon degraders (i.e., *Desulfotignum* species) (Harms et al., 1996; Ommedal and Torsvik, 2007; Higashioka et al., 2011; Abu Laban et al., 2015; Almstrand et al., 2016; Daghio et al., 2016).

Notably, recent studies reported the occurrence of *Desulfobulbaceae* members in sulfidic rich and current-producing sediments (Nielsen et al., 2010; Pfeffer et al., 2012; Daghio et al., 2016). These microorganisms form an electron transporter filamentous-like structure composed by long cables containing thousands of cells that share an outer membrane serving as electrical insulation from the external medium. This structure allows the establishment of an electron-conducting system through the sediment able to directly connect the sulfide-oxidation in the suboxic zone with the oxygen-reduction at the oxic zone (Nielsen et al., 2010; Roden et al., 2010; Pfeffer et al., 2012; Kato, 2016). As shown in Figure 1, the massive occurrence in the marine sediment of similar filamentous bacteria belonging to *Desulfobulbaceae* was found.

Despite *Desulfobulbaceae* are commonly known as sulfate reducing bacteria living in the ocean floor where the deeper cells do not have access to the oxygen, some studies also reported that the deeper cells might initiate hydrogen sulfide oxidation to elemental sulfur with oxygen serving as a spatially distant electron acceptor. These strategies allow sulfate-reducing bacteria, while inhabiting anoxic environments, to compete with other aerobic sulfide oxidizing bacteria (Fuseler et al., 1996; Finster, 2008). Moreover, some *Desulfobulbaceae* members (i.e., *Desulfobulbus, Desulfofustis, Desulfocapsa* species) were recently distinguished for their ability to couple growth to the disproportionation of elemental sulfur to sulfate and sulfide (Pagani et al., 2011; Abu Laban et al., 2015).

Within *Proteobacteria*, some *Gammaprotebacteria* were also found in the contaminated marine sediment, such as *Sedimenticola* spp. (<1% of total OTUs), most of them known as sulfur-oxidizing bacteria in marine environments capable of coupling the oxidation elemental sulfur and sulfide to autotrophic growth and to produce sulfur inclusions as metabolic intermediates (Flood et al., 2015).

Hydrocarbon petroleum biodegradation was also likely sustained by other anaerobic hydrocarbon degraders affiliated to *Anaerolineaceae* family (*Chloroflexi* phylum) comprising obligate anaerobes, whose presence has been already documented in many hydrocarbon environments, including marine sediments, where biodegradation of oil-related compounds occurred (Sherry et al., 2013; Liang et al., 2015). In line with our observations, *Anaerolineaceae* have been also reported as fundamental community members in metabolism of low-molecular-weight alkanes under sulfate-reducing conditions (Savage et al., 2010). As well as for the yet uncultured *Desulfobulbaceae* OTUs retrieved in the marine sediment, a detailed taxonomic affiliation of *Anaerolineaceae* OTUs was not reached by NGS analysis and surely further investigations will be necessary to better define the taxonomy and the physiology of these microorganisms. Further, a remarkable presence of *Deferribacteres* members in the sediment was observed. They mostly belonged to SAR406 clade (Marine group A), recently named “*Marinimicrobia*” and known to be ubiquitously distributed in oxygen minimum zones of marine environments (Stevens and Ulloa, 2008; Schattenhofer et al., 2009). Members of the phylum *Deferribacteres* were shown to be able to respire anaerobically different organic substrates by using Fe^+3^, Mn^+4^, S^0^, Co^+3^, or nitrate as electron acceptors. Interestingly, previous studies reported the occurrence of *Deferribacteres* members in oil contaminated submarine anoxic zones where they have nitrogen fixing ability and are also able to utilize a variety of both complex organic compounds or small molecules substrates, like hydrogen and acetate, as electron donors (Greene et al., 1997; Wang et al., 2011; Liang et al., 2015; Yilmaz et al., 2015).

Even though the metabolism of these microorganisms is poorly understood, the occurrence of *Deferribacteres* members deserves attention as they might have a role in the anaerobic petroleum biodegradation in contaminated marine sediments and future efforts should be addressed to elucidate the role of these microorganisms in such polluted environments.

### Snorkel-colonizing microorganisms

The electrode surface was remarkably colonized by *Alphaproteobacteria*, mostly affiliated to *Rhodospirillaceae* family, whose members belong to unidentified genera (12% of total OTUs) or to *Magnetovibrio* genus (11% of total OTUs). *Rhodospirillaceae* are purple non-sulfur bacteria able to photoassimilate anaerobically simple organic compounds. Some genera grow photoheterotrophically under anoxic conditions in the light and chemoheterotrophically in the dark, while others grow heterotrophically under aerobic and microaerophilic conditions.

Interestingly, recent studies have reported the isolation of some *Rhodospirillaceae* species from contaminated marine environments exhibiting hydrocarbonoclastic potential under anaerobic conditions in bioelectrochemical remediation systems (Venkidusamy and Megharaj, 2016). This may indicate a role in the anaerobic petroleum hydrocarbon biodegradation of *Rhodospirillaceae* species living tightly to the electrode surface.

Considerably, *Rhodospirillaceae* members are also known as magnetotactic bacteria (MTB), microorganisms present at the oxic-anoxic transition zone where opposing gradients of oxygen and reduced sulfur and/or iron exist (Geelhoed et al., 2010). They are able to biomineralize a unique organelle, called magnetosome displaying polar magnetotaxis, where magnetic iron mineral crystals are formed (Lefèvre and Wu, 2013; Barber-Zucker and Zarivach, 2016; Lefèvre, 2016). This capability probably originated as a result of the toxicity of free iron in the cells (Lefèvre and Wu, 2013). These metabolic features of *Rhodospirillaceae* are in line with previous observations regarding the formation of a Fe^3+^ reddish biofilm on the electrode surface of the Oil Spill Snorkel system (Cruz Viggi et al., 2015). Probably the colonization of *Rhodospirillaceae* carrying magnetotactic abilities is linked to the availability of the magnetosome precursors (i.e., Fe^3+^) on the electrode surface.

Further, previous studies reported members of *Rhodospirillaceae* being able to oxidize reduced sulfur species (e.g., sulfide or thiosulfate) to sulfate using oxygen as terminal electron acceptor, under microaerophilic conditions (Geelhoed et al., 2010). Possibly, in the Oil Spill Snorkel microcosms, whereby anaerobic conditions prevailed, these microorganisms thrived using the electrode (in place of oxygen) as terminal electron acceptor for the oxidation of sulfide to sulfate. This could explain the apparently lower sulfate reducing activity observed in the Snorkel treatments compared to the Snorkel-free controls (Cruz Viggi et al., 2015).

In detail, NGS data showed that a large portion of *Rhodospirillaceae* members found at the electrode surface was mainly affiliated to *Magnetovibrio* genus. Representatives of this genus are MTB bacteria and were isolated from sulfide-rich sediments. They are able to grow chemoheterotrophically with organic and some amino acids as carbon and electron source or chemoautotrophically on thiosulfate and sulfide with oxygen as terminal electron acceptor (microaerophilic growth) and on thiosulfate using nitrous oxide (N_2_O) as terminal electron acceptor (anaerobic growth) (Bazylinski et al., 1988, 2013; Lefèvre and Wu, 2013). Moreover, *Rhodospirillaceae* members other than *Magnetovibrio* such as chemoheterotrophic facultative anaerobic genera (i.e., *Nisaea*), strictly aerobic and microaerophilic genera (i.e., *Thalassospira*) were also found on the electrode surface. In particular, some *Thalassospira* strains have been isolated and sequenced as electrogenic petroleum-degrading bacteria (Kiseleva et al., 2015). Moreover, marine sediment-derived strains were reported to exhibit electrotrophic behavior, accepting electrons from insoluble sulfur but the capacity of these strains to transfer electrons to an anode has not already proven (Rowe et al., 2015). Overall, the presence of these microorganisms might suggest the occurrence of the anaerobic/aerobic gradient through the graphite electrode buried in the sediment, which allows the creation of a bioelectrochemical connection between the anoxic sediment and the overlying oxic water, driving the oxidation of petroleum hydrocarbons (to carbon dioxide and water) or reduced sulfur species such as elemental sulfur, sulfide, or thiosulfate (to sulfate).

Interestingly, sequences belonging to *Defluviicoccus* genus, commonly detected in anaerobic-aerobic wastewater treatment plants (Lanham et al., 2008; Burow et al., 2009), were obtained from the electrode surface. Some recent studies reported the occurrence of members of this genus in marine environments like cold-water coral reefs as hotspots of carbon mineralization (Van Oevelen et al., 2009; Rovelli et al., 2015; Van Bleijswijk et al., 2015).

Besides the massive presence of *Alphaprotebacteria*, sulfur oxidizing *Sedimenticola* members of *Gammaproteobacteria* (Flood et al., 2015) were also found at the electrode surface. Moreover, a large portion of unidentified OTUs was retrieved suggesting the need of further efforts to shed light on the identity of novel microorganisms involved in petroleum hydrocarbons bioelectrochemical degradation in marine environments.

### Grasping the system as a whole: biological network between the marine sediment and the electrode

**Figure 4:**
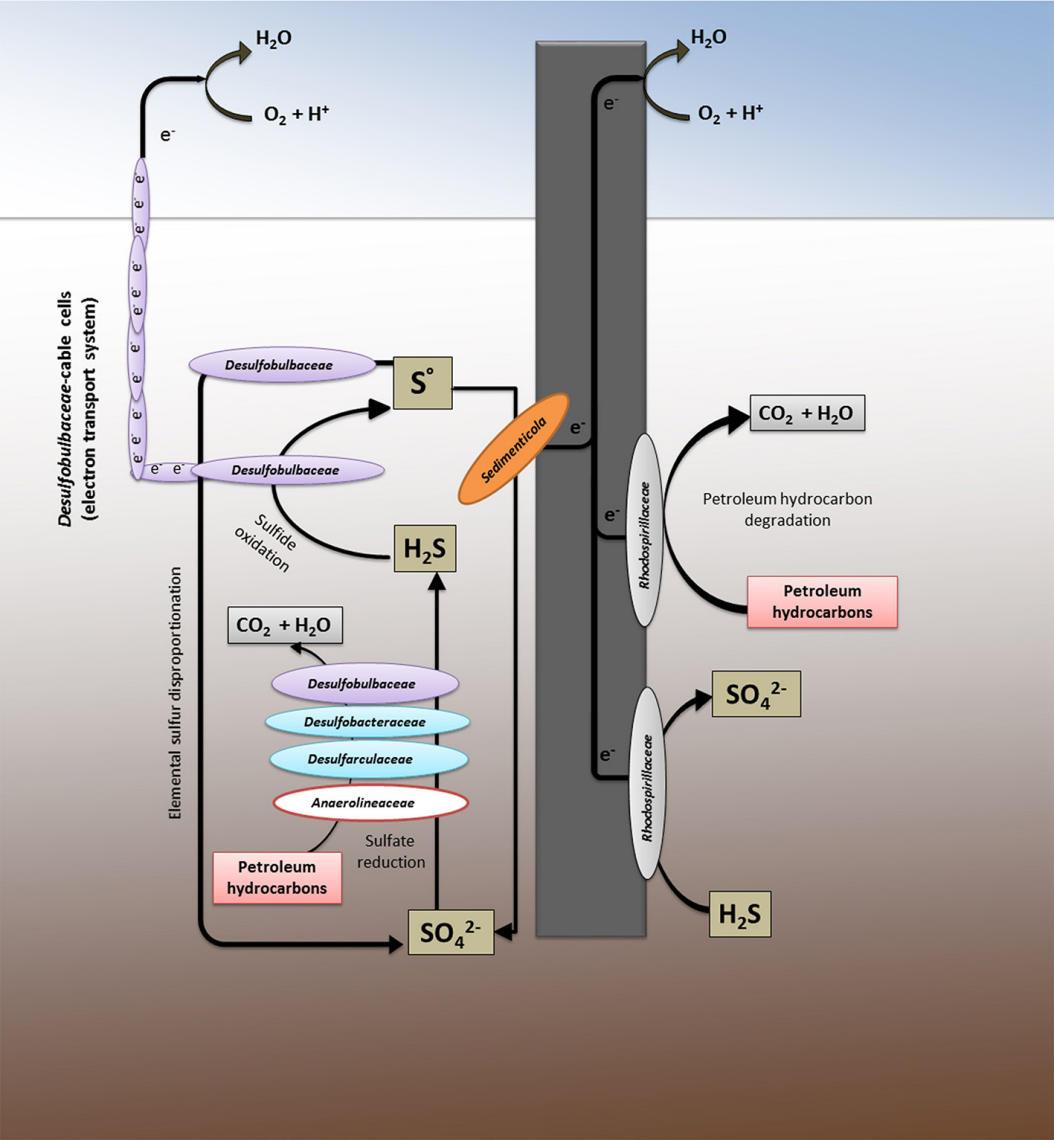
The system as a whole: the metabolic network between the marine sediment and the graphite electrode.

A tentative model of the biological network found in the contaminated marine sediment and at the electrode surface is schematically shown in Figure 4. Our findings suggest the existence in the Oil Spill Snorkel system of two parallel electrical cables, the artificial (graphite electrode) and the natural (*Desulfubulbaceae* filaments) electron conduits, which stimulate the hydrocarbons biodegradation through the establishment of an efficient sulfur cycling mediated by multiple interconnecting metabolic pathways.

Petroleum hydrocarbon biodegradation occurs in the contaminated marine sediment primarily via sulfate reduction, being sulfate the main oxidizing agent in the marine reaction environment. This process is efficiently sustained by the oxidation of sulfide to inorganic sulfur mediated simultaneously by the graphite electrode and by the cable bacteria, both capable to vehicle electrons from hydrogen sulfide, resulted from sulfate reduction in the anoxic sediment, to oxygen as a spatially distant electron acceptor. The further sulfur oxidation, driven by the electrode and mediated by several bacteria (e.g., members of *Desulfubulbaceae, Sedimenticola*, and *Rhodospirillaceae*), as well as likely the sulfur disproportion to sulfate, may regenerate sulfate in the sediment allowing to the sulfur cycle to start over again. This finding is in line with the observation of an apparently lower sulfate reduction observed in the sediment containing the electrodes compared to the control, tentatively previously linked to a back-oxidation of sulfide to sulfate (Cruz Viggi et al., 2015).

Whereas microbes affiliated to *Deltaproteobacteria* drive most of the biological processes in the sediment, analog reactions at the electrode or in the proximity of the electrode are controlled mainly by *Alphaproteobacteria* (mostly members of *Rhodospirillaceae*). The latter family contains microbes with high metabolic versatility including magnetotactic bacteria affiliated to *Magnetovibrio* genus which are often reported to occur in the aerobic-anoxic transition zone in water or sediment where opposing gradients of oxygen and reduced sulfur and/or iron exist.

Overall, the picture defined by NGS analysis showed the occurrence in the system of a variety of electroactive microorganisms able to sustain the contaminant biodegradation alone or by means of an external conductive support through the establishment of a bioelectrochemical connection between two spatially separated redox zones and the preservation of an efficient sulfur cycling. This potential might be higher than the one here described due to the unexplored identity and physiology of many OTUs generated by NGS analysis.

## Author contributions

All authors contributed equally to this work. BM performed the biomolecular experiments, analyzed data and wrote the paper. CCV and FA constructed the Oil Spill Snorkel system. SR conceived and coordinated the study and wrote the paper. All authors reviewed the results and approved the final version of the manuscript.

### Conflict of interest statement

The authors declare that the research was conducted in the absence of any commercial or financial relationships that could be construed as a potential conflict of interest.
